# Rare disruptive mutations in ciliary function genes contribute to testicular cancer susceptibility

**DOI:** 10.1038/ncomms13840

**Published:** 2016-12-20

**Authors:** Kevin Litchfield, Max Levy, Darshna Dudakia, Paula Proszek, Claire Shipley, Sander Basten, Elizabeth Rapley, D. Timothy Bishop, Alison Reid, Robert Huddart, Peter Broderick, David Gonzalez de Castro, Simon O'Connor, Rachel H. Giles, Richard S. Houlston, Clare Turnbull

**Affiliations:** 1 Division of Genetics and Epidemiology, The Institute of Cancer Research, London SM2 5NG, UK; 2 Centre for Molecular Pathology, The Royal Marsden NHS Foundation Trust, London SM2 5NG, UK; 3 Department of Nephrology and Hypertension, Regenerative Medicine Center Utrecht, University Medical Center Utrecht, Uppsalalaan 6, Utrecht 3584CT, The Netherlands; 4 Section of Epidemiology and Biostatistics, Leeds Institute of Cancer and Pathology, Leeds LS9 7TF, UK; 5 Academic Radiotherapy Unit, The Institute of Cancer Research, London SM2 5NG, UK; 6 Centre for Cancer Research and Cell Biology, Queen's University Belfast, Belfast BT9 7AE, UK; 7 Division of Molecular Pathology, The Institute of Cancer Research, London SM2 5NG, UK; 8 William Harvey Research Institute, Queen Mary University, London EC1M 6BQ, UK; 9 Department of Clinical Genetics, Guy's and St Thomas' NHS Trust, London SE1 9RS, UK

## Abstract

Testicular germ cell tumour (TGCT) is the most common cancer in young men. Here we sought to identify risk factors for TGCT by performing whole-exome sequencing on 328 TGCT cases from 153 families, 634 sporadic TGCT cases and 1,644 controls. We search for genes that are recurrently affected by rare variants (minor allele frequency <0.01) with potentially damaging effects and evidence of segregation in families. A total of 8.7% of TGCT families carry rare disruptive mutations in the cilia-microtubule genes (CMG) as compared with 0.5% of controls (*P*=2.1 × 10^−8^). The most significantly mutated CMG is *DNAAF1* with biallelic inactivation and loss of *DNAAF1* expression shown in tumours from carriers. *DNAAF1* mutation as a cause of TGCT is supported by a *dnaaf1*^*hu255h*^(+/−) zebrafish model, which has a 94% risk of TGCT. Our data implicate cilia-microtubule inactivation as a cause of TGCT and provide evidence for CMGs as cancer susceptibility genes.

## Abstract (web summary)

There is some evidence of a hereditary risk for developing testicular germ cell tumours. Here, the authors use whole-exome sequencing and identify a risk variant for the disease in *DNAAF1*, a gene involved in microtubule-based cilia.

## Graphical abstract

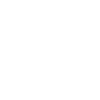

Testicular germ cell tumour (TGCT) is the most common cancer in men aged 15–45 years, with over 18,000 new cases diagnosed annually in Europe^1^,^2^,^3^. Cure rates for TGCT are typically high because of the extreme sensitivity of malignant testicular germ cells to chemotherapy; however, an increased risk of metabolic syndrome, infertility and secondary cancer associated with survivorship is now recognized^4^,^5^,^6^. Furthermore, there are limited options for patients with platinum-resistant tumours, for whom long-term survival remains poor. It is therefore anticipated that an increased understanding of TGCT pathogenesis will generate new therapeutic targets.

Family, twin and migrant studies support a strong inherited genetic basis to TGCT susceptibility, with brothers of cases having a four- to eightfold increased risk of TGCT^7^,^8^,^9^,^10^. While Mendelian susceptibility to TGCT has been inferred from the combination of the high familial risks and reports of multiplex TGCT families, no rare high-impact alleles have so far been identified^11^. In contrast to associations identified through genome-wide association studies, the identification of this class of susceptibility is especially important since such mutations are causal and thus provide direct insight into TGCT biology.

Here, we use whole-exome sequencing (WES) of germline DNA to identify novel high-impact TGCT risk variants, focusing our analysis on familial TGCT as such cases are enriched for genetic susceptibility. We identify rare disruptive mutations in cilia-microtubule function genes as determinants of susceptibility to TGCT. This is further supported by evidence of second somatic mutation in tumour tissue and functional data from zebrafish, which collectively suggest a model of cilia inactivation in promoting TGC tumorigenesis.

## Results

### Whole-exome sequencing of familial TGCT cases

**Figure 1:**
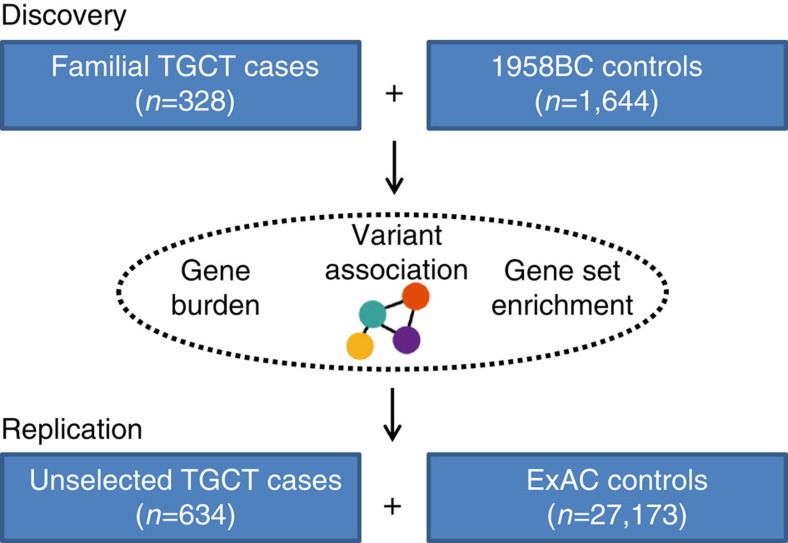
Study design. Overview of patient samples and exome sequencing study design.

To identify rare germline variants involved in TGCT we performed WES of 328 TGCT cases from 153 independent families of European ancestry (Methods). For comparison we analysed WES data on 1,644 UK population controls from the 1958 Birth Cohort (1958BC) with no personal history of malignancy (Fig. 1). DNA from germline blood samples from cases and controls was sequenced using Illumina TruSeq exon capture or Nextera Rapid Capture in conjunction with Illumina Hi-Seq 2000 or 2500 technology (Methods). To avoid erroneous findings we performed alignment and variant calling of all samples simultaneously (Methods). Each captured base was sequenced to an average depth of 49 × across samples.

### Gene burden analysis of familial TGCT data

**Figure 2:**
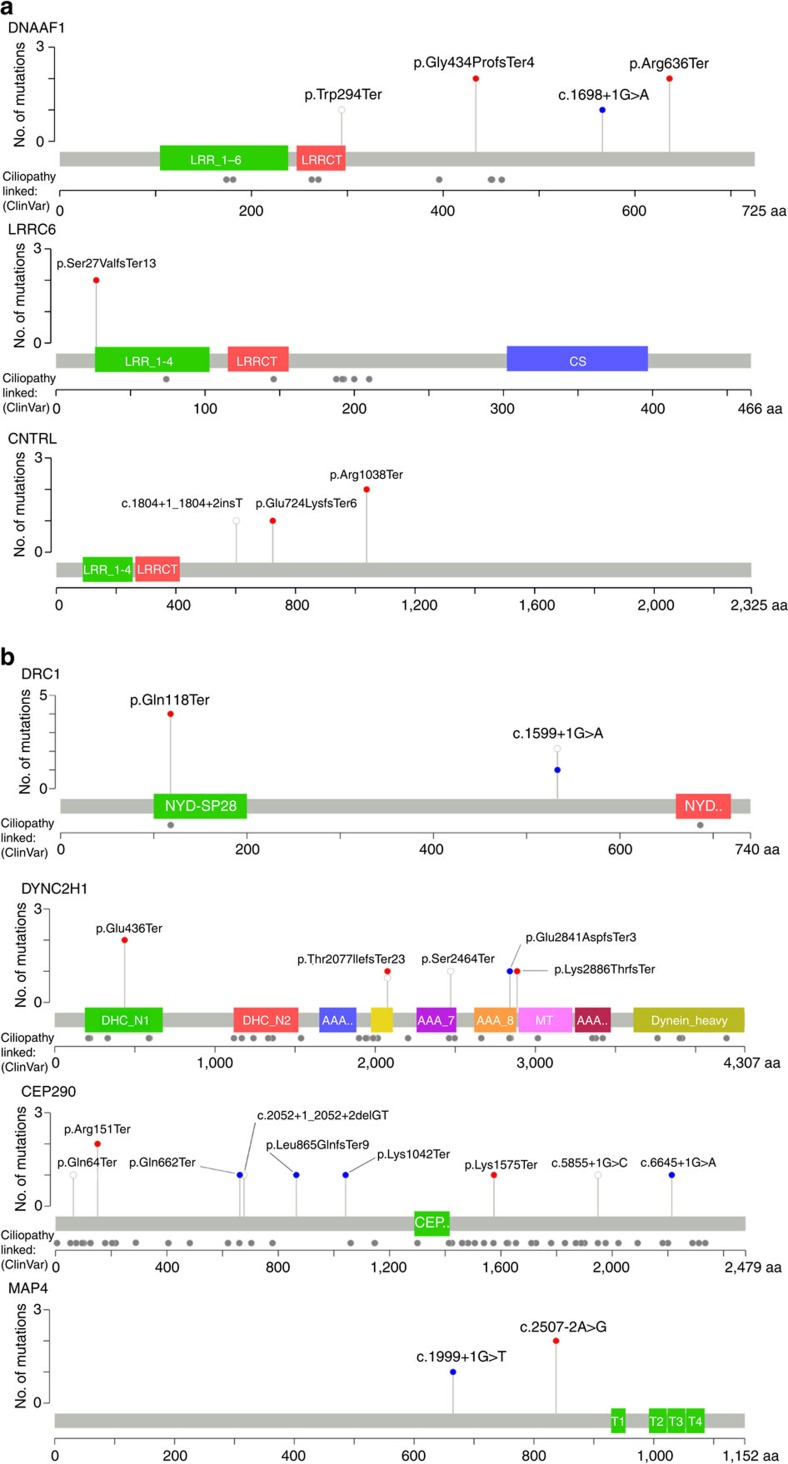
Disruptive germline mutations in cilia-microtubule pathway genes identified in TGCT cases. (**a**) DNAAF1 and paralogue genes; (**b**) cilia-microtubule gene set. Red dots denote mutations identified in familial TGCT cases, blue dots denote mutations in unselected TGCT cases and white dots denote mutations in UK controls. Grey dots denote mutations catalogued by ClinVar^24^ as a cause of recessive ciliopathy. Domain abbreviations: LRR, leucine-rich repeat; LRRCT, leucine-rich repeat C-terminal; CS, CHORD-containing proteins and SGT1; NYD-SP28, NYD-SP28 sperm tail; NYD.., NYD-SP28_assoc sperm tail C-terminal domain; DHC_N1, dynein heavy chain, N-terminal region 1; DHC_N2, dynein heavy chain; N-terminal region 1, AAA.., hydrolytic ATP-binding site of dynein motor region D1; AAA_7, P-loop containing dynein motor region D3; AAA_8, P-loop containing dynein motor region D4; MT, microtubule-binding stalk of dynein motor; Dynein_heavy pfama, dynein heavy chain and region D6 of dynein motor; CEP.., coiled-coil region of centrosome protein; M1-4, Tau/MAP 1–4.

**Figure 3:**
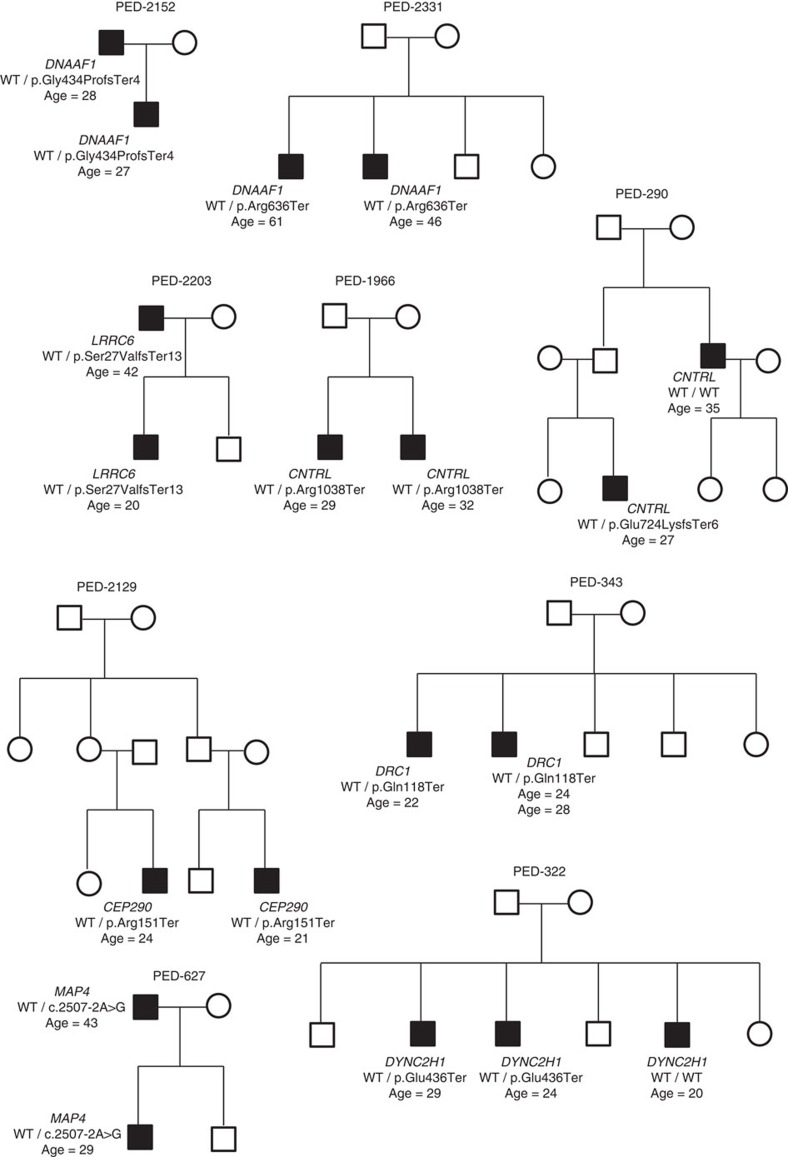
Segregating TGCT pedigrees of cilia-microtubule pathway gene carriers. Circles, female; and squares, male. TGCT cases denoted by shaded symbols; ages refer to age at diagnosis of TGCT.

We searched in familial cases for genes that were recurrently affected by rare variants with presumptive damaging effects (nonsense, splice acceptor/donor and indel frameshift changes) that had a low burden of comparable variants in controls. We did not include potentially damaging missense variants because of the limited accuracy of *in silico* prediction tools in predicting pathogenicity of missense variants for human disease (American College of Medical Genetics^12^). We performed a collapsing T1 gene burden test imposing a maximal minor allele frequency (MAF) threshold of 1%, to select for rare high-impact variants. To ensure independent events, case counts were based on one individual per pedigree, which was randomly assigned as the proband. Significance was assessed by permutation. To prioritize genes for high-impact variants we filtered results to select only genes containing rare disruptive mutations which segregated with TGCT in at least two families (Table 1). There was no gene for which mutations were detected in more than three of the 153 families when the filters described for mutation type, frequency and segregation were applied. The top ranked gene exome-wide was *DNAAF1/LRRC50 (*Dynein, Axonemal, Assembly Factor 1/Leucine-rich repeat containing 50), with the rare disruptive mutations p.Arg636Ter and p.Gly434ProfsTer4 (Fig. 2a) segregating with TGCT in PED-2331 and PED-2152 families (Fig. 3). *DNAAF1* forms a component of the microtubule outer dynein arm, stabilizing microtubule-based cilia^13^. A deleterious phenotype in humans for disruptive *DNAAF1* mutations has previously been established, with biallelic mutations causing recessive primary ciliary dyskinesia (PCD)^14^, which is characterized by impaired primary cilia function, chronic lung disease, male infertility and hearing impairment^15^,^16^. In addition to *DNAAF1,* mutations in the paralogue genes *LRRC6* (ref. 17) and *CNTRL* (centriolin; ref. 18) were identified in TGCT cases in three additional families. In total *DNAAF1* and its paralogues were mutated in nine cases, from five families with segregation of mutations detected in four families (Fig. 3).

### Gene set enrichment analysis of familial TGCT data

To complement our T1 burden analysis of single genes, we conducted a Gene-Set-Enrichment-Analysis based on the GO Biological Process ontology in order to identify groups of genes based on specified biological processes/pathways associated with TGCT the signal for which would not have individually been detectable in exome-wide analysis. The top ranked set from analysis of 1,090 canonical gene sets was the gene set related to cilia-microtubule function (*P*=2.1 × 10^−8^, *Q*=0.01, permutation test), containing genes *DNAAF1, DYNC2H1, DRC1, CEP290* and *MAP4* (Table 2 and Supplementary Table 1) which are each a cause of recessive ciliopathy (PCD—*DNAAF1*/*DRC1* (refs 14,19); asphyxiating thoracic dystrophy—*DYNC2H1* (ref. 20); Joubert syndrome—*CEP290* (refs 21, 22); and Senior-Loken syndrome—*MAP4* via *TRAF3IP1* mutation^23^). Moreover, even excluding *DNAAF1*, the cilia-microtubule gene set remained the most significantly associated of the 1,090 gene sets (*P*=9.1 × 10^−6^, *Q*=0.1, permutation test). The mutations we identified in the TGCT cases cluster predominantly in the same ciliopathic-associated protein domains as mutations causing the recessive diseases (Fig. 2b)^24^.

### Replication sequencing of unselected TGCT cases

We next performed WES of 634 sporadic TGCT cases from the UK, comparing mutation frequencies with 27,173 ExAC non-Finnish European, cancer-free controls^25^. In gene-set enrichment analysis (GSEA), association was replicated in this data set for the cilia-microtubule gene set (*P*=0.024, Fisher's Exact Test), with additional rare disruptive case variants identified in *DNAAF1*, *MAP4, DRC1, DYNC2H1* and *CEP290*. Remarkably as well as an additional *DNAAF1* mutation, an additional *MAP4* mutation was observed, taking the total number of *MAP4* rare disruptive case mutations to three; whereas no rare disruptive mutations in *MAP4* were observed in 27,173 ExAC controls (*P*=1.9 × 10^−5^, Fisher's Exact Test), the 1,644 UK controls or 4,300 European controls from the exome variant server project.

### Somatic alterations in mutation carriers

**Figure 4:**
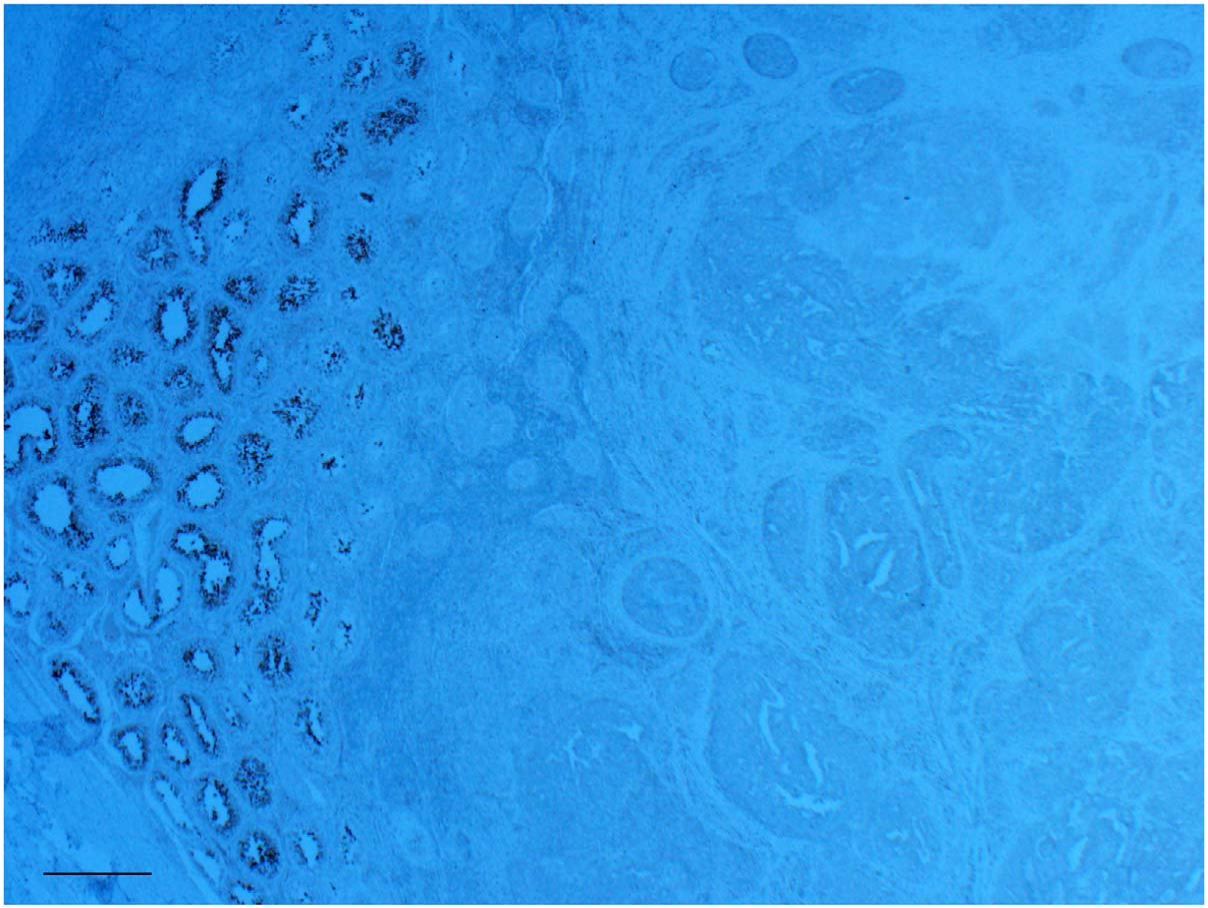
IHC staining for DNAAF1 expression in available tumour tissue from mutation carriers. IHC showing positive DNAAF1 expression in surrounding normal tissue (left) but loss of expression within the tumour (middle and right). Data are shown for tumour from PED-2152 (p.Gly434ProfsTer4). A comparable pattern was found in PED-2331 (p.Arg636Ter) and S-1645 (c.1698+1G>A). Scale bar, 5 μm.

Immunohistochemistry (IHC) staining for DNAAF1 showed complete absence in 3/3 tumours available from *DNAAF1* mutation carriers, with presence of the protein demonstrable in normal surrounding tissue (Fig. 4). Evidence of inactivation of the second allele was also demonstrated on sequencing of two of the three tumours (Supplementary Fig. 1). Collectively these findings are compatible with *DNAAF1* having a tumour suppressor function.

### Functional studies in zebrafish model

We have previously implicated mutation of *DNAAF1* as a cause of TGCT in zebrafish (*n*=30) with loss of heterozygosity of *DNAAF1* demonstrated in the tumours^26^. To further explore the link between disruptive mutations in *DNAAF1*, ciliary function and TGCT, we conducted additional studies in *dnaaf1*^*hu255h*^ mutant and wild-type zebrafish. We first examined the frequency and characteristics of TGCTs in 136 heterozygotely mutated *dnaaf1*^*hu255h*^ male zebrafish compared with 114 age-matched male wild-type fish: TGCT were observed in 94% (128/136) of *dnaaf1*^*hu255h*^ (+/−) mutants as compared with 14% (16/114) of those with wild-type genotype (*P*=3.5 × 10^−14^, Fisher's Exact Test) (Supplementary Fig. 2a). Tumours were characterized by severely reduced end-stage differentiated germ cells and an increase in early spermatogonial-like cells thus closely resembling human seminoma (Supplementary Fig. 2b). We have previously extensively demonstrated a morphological and histological analogy of zebrafish TGCTs to human TGCTs (Supplementary Fig. 2b), specifically for the subtype seminoma, based on established markers for early spermatogonia and spermatozoa. Nevertheless, despite the recognized similarities between the genera, we acknowledge there are discernible differences in gametogenesis between zebrafish and human germ cells, including distinct sex determination mechanisms of the germ cell lineage and markedly different architectural composition of the gonads in terms of somatic stem cell niche presence and a cystic expansion versus progressive tubular differentiation, respectively.

To explore the potential mechanism by which cilia function promotes TGCT formation, electron microscopy of spermatogonia intercellular bridges in wild-type fish was performed. We noted that the intercellular bridges of early spermatogonia in wild-type zebrafish are flanked by ciliary structures (Supplementary Fig. 3), suggesting a role for cilia in spermatogonial stem cell differentiation, the failure of which is considered to be a fundamental precursor step in TGCT oncogenesis. This observation is also consistent with a model whereby impaired cilia function, for example through loss of *DNAAF1*, triggers structural instability preceding premature dissociation of early germ cells and concomitant differentiation disruption. Further functional studies are needed to confirm this hypothesis.

### Analysis of the cancer genome atlas data

More than 40% of cancer susceptibility genes are found to be tumorigenic when mutated only in tumour DNA^27^, accordingly we sought to assess whether *DNAAF1* was also frequently lost somatically. Analysis of 150 human TGCTs publically available through the cancer genome atlas project (http://cancergenome.nih.gov/) showed significant focal somatic deletion at 16q23-16q24.3 (*Q*=1.6 × 10^−4^, from GISTIC2 with significance assessed by permutation, corrected for multiple testing using the Benjamini–Hochberg method), encompassing *DNAAF1,* is a feature in 24.7% of tumours, which predominantly have seminoma histology. In addition *DNAAF1* is highly methylated in seminomas, as compared with non-seminomas (*P*=2.3 × 10^−7^, Kruskal–Wallis test), and this is accompanied by downregulation of *DNAAF1* expression as compared with normal testis (*P*=2.2 × 10^−16^, Kruskal–Wallis test). Intriguingly all of the five cases we identified with rare germline *DNAAF1* mutation had either seminoma (*n*=4) or mixed histology (*n*=1). Collectively these data are consistent with loss of *DNAAF1* having a more general impact on seminoma oncogenesis.

## Discussion

Here we report the largest WES study to date of familial and sporadic TGCT, identifying rare mutations in CMGs as determinants of disease susceptibility. A pertinent question relates to the overlapping phenotypic impact of CMG mutation, with the genetic and functional data presented here linking heterozygote mutation to TGCT risk, while homozygote mutations have been previously associated with a range of rare autosomal recessive ciliopathies. While we have identified descriptive case reports of TGCT coincident with PCD^28^,^29^, a robust statistical assessment of disease coincidence is not currently possible due to the rare nature of both conditions and the lack of systematic registry data. A model whereby heterozygote but not homozygote CMG mutation is primarily associated with TGCT risk is reconcilable, owing to the importance of timing in TGCT oncogenesis, with proliferative growth occurring in a specific post-pubertal time window. Under such a model, biallelic loss of function present at birth may trigger differing phenotypic consequences to heterozygote germline mutation followed by a second later somatic event. Finally infertility features as a common factor associated with both TGCT and PCD^16^,^17^, further supporting a potential phenotypic relationship, and while we cannot discount infertility as an intermediate phenotype promoting TGCT risk, the combined human and zebrafish rare deleterious mutational data strongly support genetic causality. In terms of functional mechanisms, we note that loss of cilia function is emerging more broadly as an important pathway in tumorigenesis in multiple cancer types^30^,^31^,^32^. The functional basis of inactivation of CMGs in oncogenesis remains to be established; however, ciliation and the cell cycle are mutually exclusive with both processes competing for the centrosome^33^. Hence cilia inactivation may bias towards cell cycle progression and proliferative growth.

The focus of our study was on disruptive, protein truncating mutations; with missense variants not included on account of insufficient tools to reliably predict pathogenicity^12^. This issue is exemplified by a previous study of *DNAAF1* (ref. 26), in which candidate missense variants in human TGCT cases were proposed as pathogenic; case–control analysis of these variants confirms that they are of equivalent frequency between cases and control series (Supplementary Table 2). In the broader context of understanding the genetic architecture of TGCT, while providing evidence for the role of rare variants as risk factors, our analysis is consistent with the previously proposed model of polygenic susceptibility, in which much of the heritable risk of TGCT is associated with common genetic variants^34^. Such a model is supported by the recent GWAS which have so far identified 25 risk loci which collectively account for 19% of the familial TGCT risk^34^,^35^,^36^,^37^,^38^,^39^,^40^,^41^,^42^,^43^,^44^,^45^,^46^,^47^.

In conclusion, we have provided evidence for the role of inherited mutations in CMGs as determinants of TGCT, identifying germline disruptive mutations in 9% of familial pedigrees, with additional evidence implicating disruptive mutations in *DNAAF1* in TGC tumorigenesis from IHC and sequencing studies of human tumours and a *dnaaf1*^*hu255h*^ (+/−) zebrafish model displaying a 94% frequency of TGCT. As well as revealing insights into disease pathways, our data provides a resource for contextualizing the impact of future candidate TGCT genes.

## Methods

### Ethics

Written informed consent was obtained from all individuals with ethical review board approval (UK National Cancer Research Network Multi-Research Ethics Committee—MREC02/06/66, 06/MRE06/41) and the study was conducted in accordance with the declaration of Helsinki.

### Subjects and data sets

TGCT cases were ascertained from (i) ‘The UK Genetics of Testicular Cancer Study' and (ii) ‘Identification, epidemiological and molecular analyses of families with susceptibility to TGCT' (recruitment via the UK Testicular Cancer Collaboration and International Testicular Cancer Linkage Consortium; Supplementary Notes 1 and 2) which were coordinated by The Institute of Cancer Research. All cases had self-reported European ancestry. Of the 328 familial cases 289 were of UK origin, and 39 were of non-UK European ancestry. All 634 sporadic cases were from the UK. The controls comprised 1,644 healthy individuals from the UK 1958 Birth Cohort^48^—974 from the ICR1000 data set (EGAD00001001021) and an additional 670 individuals (EGAS00001001667) also sequenced at Institute of Cancer Research under the same protocol.

### Whole-exome sequencing

1ug of DNA from each individual was fragmented using a Covaris E Series instrument (Covaris Inc. Woburn, MA, USA). Indexed paired-end libraries were prepared using Illumina TruSeq 62 Mb expanded exome enrichment kit (Illumina, San Diego, CA, USA). Forty-nine samples with insufficient input DNA were prepared using Illumina Nextera Rapid Capture 37 Mb exome enrichment kit. The 2 × 100 bp sequencing was performed using Illumina HiSeq2000 or 2500 technology.

### Read mapping and variant analysis

Paired-end fastq files were extracted using CASAVA software (v.1.8.1, Illumina) and aligned to build 37 (hg19) of the human reference genome using Stampy and BWA software^49^. Alignments were processed using the Genome Analysis Tool Kit (GATKv3) pipeline according to best practices^50^,^51^. Analysis was restricted for all samples to the capture regions defined in the Truseq 62 Mb bed file plus 100 bp padding. The Variant Effect Predictor (VEP) was used to provide annotations on the predicted impact of each variant. We additionally annotated with alignability of 100mers and distance from simple repeats defined by University of California, Santa Cruz (UCSC) browser tracks. Mean coverage of 49 × was achieved across targeted bases with 83% being covered at ≥15 × ; cases and controls had similar technical sequencing metrics. We excluded 57 subjects with low-quality data (<50% of bases covered with minimum 15 × ) or non-European ancestry. An additional 21 controls samples were excluded due to sex discrepancy and detection of cancer history during the course of the analysis.

We considered only canonical transcripts and for each variant, assuming the most deleterious predicted effect for each transcript according to VEP. For all analyses we imposed GATK internal calling thresholds excluding variants as per current best practice guidelines^50^,^51^—in the 99.5th truth tranche for single-nucleotide variants and >99th tranche for indels. To minimize false positives we adopted an automated approach imposing: GQ≥30, for a heterozygous call an alternate depth ≥3 and χ^2^< 10.83 (that is, *P*>0.0001) for the observed versus expected distribution of alternate/reference alleles (alt-ref-ratio), UCSC alignability (100 bp window size)=1, not in simple repeat, Hardy–Weinberg equilibrium test (*P*>1.0 × 10^−8^) in cases and controls and an overall call rate ≥75% in both cases and controls. To eliminate false-positive associations caused by indels, genes featuring only this class of mutation type were filtered out. To ensure a high-quality variant set and reproducibility was verified for a subset of samples (*n*=162) which had also been genotyped using Illumina HumanCNV370-Duo bead arrays—>99% concordance was observed between platforms. Following QC the final data set comprised 306 familial TGCT cases (from 150 independent pedigrees), 613 unselected TGCT cases and 1,609 controls. Variants previously known to cause recessive ciliopathies (as displayed in Fig. 2) were extracted from ClinVar^24^, filtering to select only ciliopathic phenotypes and to remove variants classified as benign.

### Statistical analyses

To test whether rare mutations contribute to TGCT we performed a collapsing burden test imposing a maximal MAF threshold of 1%. To ensure independent events, case counts were based on one individual per pedigree randomly assigned as the proband. Significance levels were assessed using 10^5^ permutations on case/control status. Study power to detect was calculated using disease allele frequency in controls as the baseline allele frequency, while the frequency in cases determined by a weighted average of the enrichment found in cases with one, two and three affected first-degree relatives. Allele counts were then sampled from frequencies between 0.00001 and 0.01 and relative risks between 1.75 and 10.0. A Fisher's test was then performed for each sampling of cases and controls. This process was performed 10,000 times for each frequency/relative risk combination and for each instance the frequency of tests that were significant at an exome-wide significance of 8.0 × 10^−7^ equated to study power. Statistical analyses were carried out using R3.0.2 and Stata (v12) (StataCorp, Lakeway Drive College Station, TX, USA) software.

### Pathway analysis

To test for over-representation of damaging variants within genes mapping to a specific pathways or biological process we performed a GSEA. This comprised 1,090 canonical gene sets from KEGG, GO: biological processes and Reactome pathways, supplemented by an OMIM search-term-driven method including genes expressed in normal testicular tissue^52^ and genes with evidence of somatic inactivation from TGCT. A pre-ranked GSEA was then performed for all gene sets, with ranking of genes based on their permuted *P* values for familial cases compared with controls. For replication only the significant (*Q*<0.1) cilia-microtubule pathway was evaluated.

### Immunohistochemistry

Sections for each case were cut at 4 μm and placed on Superfrost plus slides. All slides were baked in the oven at 72 °C for 40 min before staining. Slides were stained on the Ventana BenchMark ULTRA instruments using the Ventana OptiView DAB detection kit. Slides underwent a heat mediated antigen retrieval step using Ventana Cell Conditioning 1 reagent, heating slides to 100 °C for 64 min. Following this, slides were incubated with the DNAAF1 polyclonal antibody (Abnova, catalogue number PAB23762)) for 32 min, using a dilution 1:500.

### Tumour sequencing

Rare disruptive variants in CMG found by WES were confirmed by Sanger sequencing, and examined for loss of heterozygosity in tumour samples relative to germline samples. Primers flanking variants were designed using Primer3 (ref. 53) (Supplementary Table 3). PCR amplicons of germline and tumour samples were bidirectionally sequenced using the BigDye Terminator Cycle sequencing kit and an Applied Biosystems 3730xl DNA Analyzer (Life Technologies). Sequence traces were inspected using Mutation Surveyor software (SoftGenetics, State College, PA, USA). Heterozygote variants confirmed in germline sequences were examined in corresponding tumour samples where tumour blocks were available.

### Zebrafish model

All animal experiments were ethically approved by the Animal Care Committee of the Royal Dutch Academy of Science according to the Dutch legal ethical guidelines. The human tumour samples were approved by an institutional review board (MEC 02.981). Samples were used according to the ‘Code for Proper Secondary Use of Human Tissue in the Netherlands,' developed by the Dutch Federation of Medical Scientific Societies^26^. Embryos were generated by natural pair-wise matings of heterozygous carriers and raised at 28.5 °C on a 14 h light/10 h dark cycle in a 100 mm^2^ petri dish containing aquarium water. *dnaaf1*^*hu255h*^ have a T/A mutation, changing a conserved leucine into a stop codon (L88X; ENSEMBL gene: ZGC::56169) and randomly selected control zebrafish of the same background were maintained according to standard protocols. Before tissue isolation, zebrafish were euthanized by overdose of MS222 or ice bath. Sections for IHC were fixed overnight in 4% paraformaldehyde and 2% acetic acid, embedded in paraffin and 6 μm obtained. Tissue for morphological analysis was fixed using 4% glutaraldehyde and embedded in glycol methacrylate (Technovit 7100, Hereaus Kulzer), and 4-μm sections stained with toluidine blue. Images were captured using a Nikon Eclipse E800 equipped with a Nikon DXM1200 digital camera and Plan Apo × 2/0.1, × 10/0.45, × 20/0.75 and × 40/0.95 NA objectives.

### Electron microscopy

Testes tissue was fixed in Karnovsky fixative (2% paraformaldehyde, 2.5% glutaraldehyde, 0.08M Na-cacodylate (pH 7.4), 0.25 mM calcium chloride and 0.5 mM magnesium chloride (pH 7.4)) for at least 24 h at 4 °C. Samples were postfixed in 1% osmiumtetroxide and embedded in Epon 812. Ultrathin sections (60nm) were contrasted with 3% uranyl magnesium acetate and lead citrate and viewed with a Jeol (http://www.jeol.com/) JEM 1010 or a Philips (Eindhoven, The Netherlands) CM10 transmission electron microscope.

### Analysis of The Cancer Genome Atlas data

Copy number status, RNA-Seq expression data (RPKM counts) and gene methylation data were extracted for ‘Testicular Germ Cell Tumours' from the TCGA Broad Firehose pipeline run on 28 January 2016. Normal testicular tissue RNA-Seq expression data (RPKM counts) were downloaded from GTEx^54^. Associations between differential methylation and expression levels across seminoma, non-seminoma and normal testicular tissue were quantified using the Kruskal–Wallis trend test.

### Web addresses

Genome Analysis Tool Kit (GATKv3): https://www.broadinstitute.org/gatk

Online Mendelian Inheritance in Man (OMIM): http://omim.org/

Exome Variant Server, NHLBI GO Exome Sequencing Project (ESP), Seattle, WA, USA: http://evs.gs.washington.edu/EVS (accessed January 2016)

Exome Aggregation Consortium (EXAC): http://exac.broadinstitute.org/

Gene set enrichment analysis (GSEA): http://software.broadinstitute.org/gsea/index.jsp

The cancer genome atlas (TCGA): http://cancergenome.nih.gov/

Broad Firehose: https://gdac.broadinstitute.org/

### Data availability

The WES data that supports this study have been deposited at the European Genome-phenome Archive (EGA), which is hosted by the European Bioinformatics Institute (EBI); accession numbers EGAS00001001789, EGAD00001001021 and EGAS00001001667. The TCGA data is available from the database of Genotypes and Phenotypes (dbGaP), Study Accession: phs000178.v9.p8. The remaining data are available within the article and its Supplementary Information files or available from the authors upon request.

## Additional information

**How to cite this article:** Litchfield K. *et al*. Rare disruptive mutations in ciliary function genes contribute to testicular cancer susceptibility. *Nat. Commun.* **7,** 13840 doi: 10.1038/ncomms13840 (2016).

**Publisher's note:** Springer Nature remains neutral with regard to jurisdictional claims in published maps and institutional affiliations.

## Supplementary Material

Supplementary Information Supplementary Figures, Supplementary Tables and Supplementary Notes.

## Figures and Tables

**Table 1:** Genes with rare MAF<1% disruptive mutations segregating in two or more familial TGCT pedigrees.

**Gene**	**TGCT Probands (*****n*****=150)**	**Controls (*****n*****=1,609)**	**OR**	***P*****-value**	**Total no. affected familial cases (*****n*****=306)**	**Rank**
*DNAAF1*	2	1	21.3	9.9 × 10^−3^	4	1
*ACSM1*	2	6	3.6	6.4 × 10^−2^	4	2
*TSNAXIP1*	3	11	2.9	6.9 × 10^−2^	6	3
*C1orf186*	2	8	2.7	1.3 × 10^−1^	4	4
*KIAA1586*	2	8	2.7	1.3 × 10^−1^	4	5
*ABCA10*	2	8	2.7	1.6 × 10^−1^	4	6
*PIK3C2G*	3	13	2.5	2.4 × 10^−1^	6	7
*C1orf168*	2	9	2.4	2.5 × 10^−1^	5	8
*RRP15*	3	18	1.8	2.8 × 10^−1^	6	9
*MUC4*	3	22	1.5	2.9 × 10^−1^	7	10
*OR6K2*	2	15	1.4	3.0 × 10^−1^	5	11
*KRTAP1-1*	2	15	1.4	3.0 × 10^−1^	4	12
*ABCC12*	3	23	1.4	3.3 × 10^−1^	7	13
*CALML4*	2	16	1.3	3.4 × 10^−1^	5	14

Abbreviations: MAF, minor allele frequency; OR, odds ratio; TGCT, testicular germ cell tumour.

OR calculated based on proband vs control frequency. *P*-value from T1 gene burden test, with significance assessed by permutation test.

**Table 2:** Gene set enrichment analysis results in familial TGCT data set.

**Gene set**	**Number of genes**	**Enrichment Score**	***P*****-value**	***Q*** **value**	**Rank**
Cilia-microtubule function	8	2.05	2.1 × 10^−8^	0.01	1
Reactome loss of nlp from mitotic centrosomes	27	1.93	3.1 × 10^−4^	0.11	2
Chromosome segregation	16	1.84	1.8 × 10^−3^	0.37	3
Reactome recruitment of mitotic centrosome proteins and complexes	31	1.83	6.7 × 10^−4^	0.34	4
Reactome glutathione conjugation	10	1.82	2.2 × 10^−3^	0.34	5
Response to light stimulus	23	1.81	2.1 × 10^−3^	0.33	6
Respiratory gaseous exchange	6	1.80	8.3 × 10^−4^	0.31	7
Response to radiation	29	1.78	2.8 × 10^−3^	0.37	8
Kegg abc transporters	30	1.78	2.1 × 10^−3^	0.36	9
Response to ultraviolet	15	1.76	6.1 × 10^−3^	0.41	10
Photoreceptor cell maintenance	7	1.75	3.8 × 10^−3^	0.45	11
Chromosome organization and biogenesis	41	1.74	1.7 × 10^−3^	0.46	12
Regulation of endocytosis	6	1.72	4.4 × 10^−3^	0.55	13
Reactome mitotic g2 g2 m phases	35	1.72	4.1 × 10^−3^	0.54	14
Membrane fusion	11	1.71	9.4 × 10^−3^	0.52	15

Abbreviations: TGCT, testicular germ cell tumour.

*P*-value significance assessed by permutation test.
