# Supplementary material for: Rare disruptive mutations in ciliary function genes contribute to testicular cancer susceptibility
Source: Nat Commun. 2016 Dec 20;7:13840. doi: 10.1038/ncomms13840 (PMC5187424; doi:10.1038/ncomms13840)
Supplement: Supplementary Information — Supplementary Figures, Supplementary Tables and Supplementary Notes. [file ncomms13840-s1.pdf]

Supplementary Figure 1. Evidence to support biallelic inactivation was observed in tumor material from 2/3 available cases. A 297bp region within exon 4 of DNAAF1 was captured (chr16:84203572-84203869), and germline/tumor product lengths are shown for the 2 cases with biallelic inactivation. In PED-2152 a 23bp deletion was present in this region in the germline, resulting in a germline product length of 274bp. For the PED-2152 tumor sample a second truncated product length is also clearly observed, containing a second somatic 100bp deletion not found in the germline. For S-1645, a sample from our sporadic cases, a nonsense point mutation is present in the germline, meaning a full 297 bp germline product length for this region. For the S-1645 tumor sample again a second truncated product length is detected, caused by a somatic deletion.

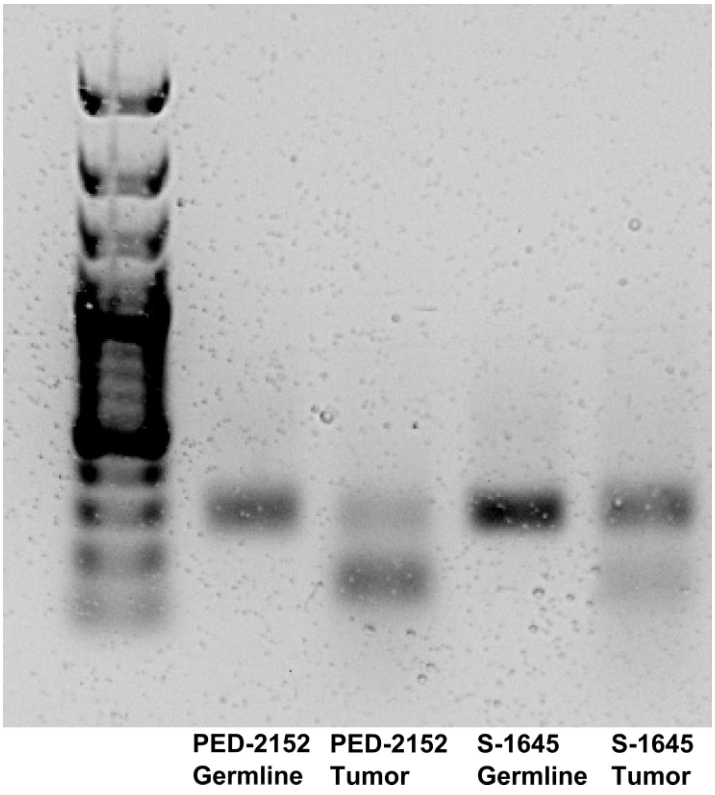

Supplementary Figure 2a. Comparison of wild-type male zebrafish and mutant *DNAAF1*^{Hu255h}(+/-) zebrafish. On top is the healthy wild-type zebrafish compared to mutant *DNAAF1*^{Hu255h}(+/-) zebrafish with visible tumor mass on bottom. TGCT was confirmed through histological characterization.

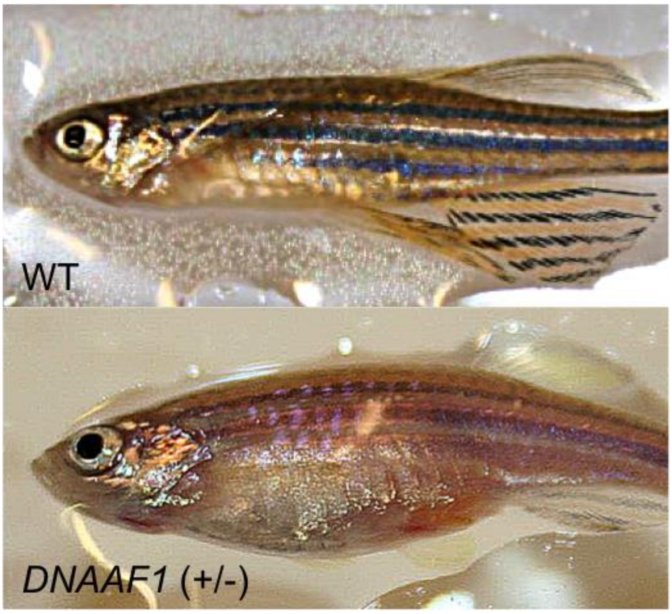

Supplementary Figure 2b. Histological characterisation of zebrafish and human tissue. On top left is age-matched (24–44 months old) wild-type zebrafish and on bottom left is the zebrafish *DNAAF1*^{Hu255h}(+/-) tumor tissue, morphological tissue analysis of toluidine blue stained sections shows a dramatic loss of differentiated germ cells. On the top right for comparison is a healthy human testis and bottom right is a human seminoma.

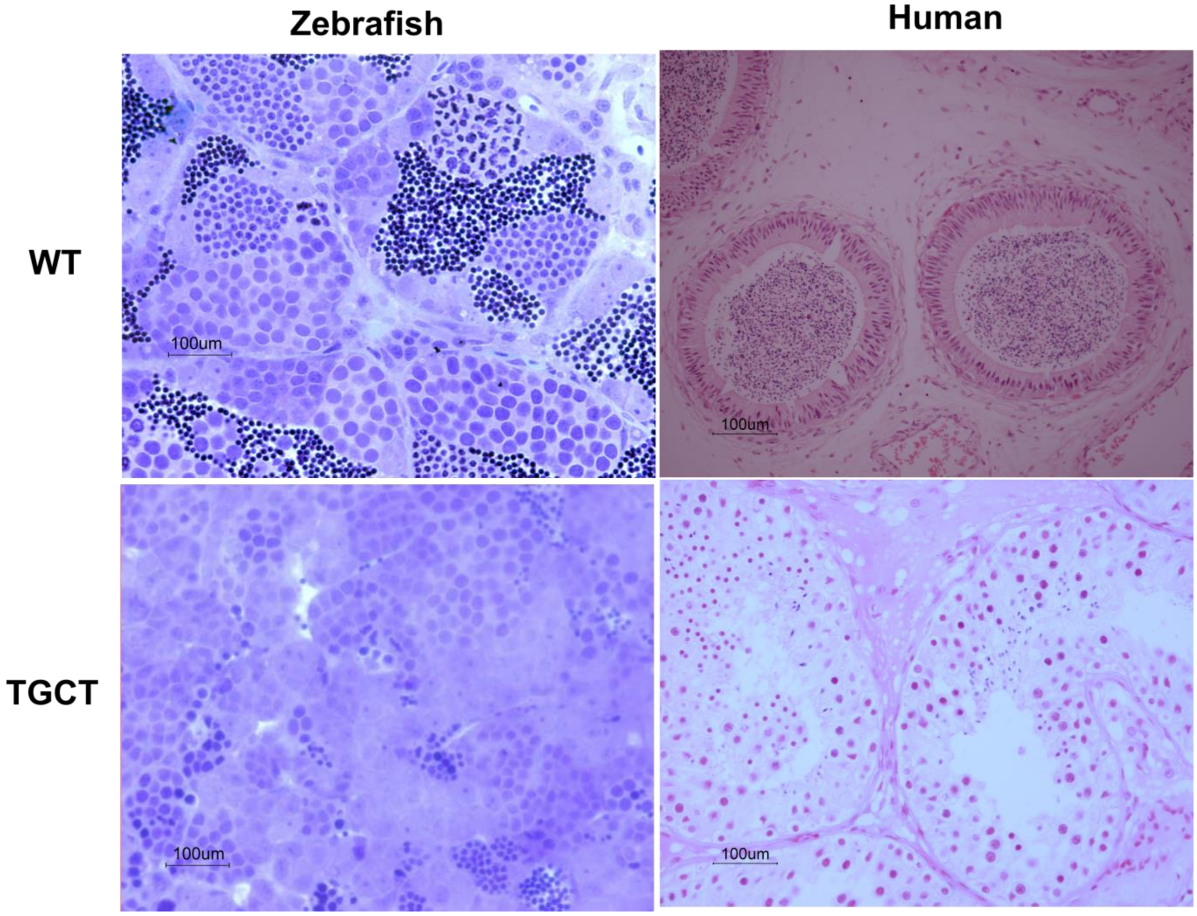

Supplementary Figure 3. Ciliary structures adjacent to germ cell syncytium intercellular bridges from high resolution images of wild-type zebrafish male gonads. Spermatogonial stem cells commit to the development of spermatozoa via sequential steps of proliferation and differentiation after initial asymmetric cell division. A hallmark feature of spermatogonial derived early germ cell is the presence of intercellular bridges, concomitant from prematurely blocked cytokinesis followed by intercellular bridge stabilization. Presented are two gonadal sections displaying two adjacent male germ cell associated through intercellular bridges. Intriguingly, we observed presence of ciliary structures in the immediate vicinity of intercellular bridges. As the sections are ultrathin, only one of either cilium is visible as the corresponding cilium or centrosome is not in the same plane. A: The lower cell 2 shows mother and daughter centrioles where microtubules emanating from the mother centriole extend into a structure reminiscent of the primary vesicle (insert**). In cell 1, one centriole can be observed in the same plane B: Cell 2 shows presence of a cilium clearly featured by an axonemal structure composed of a membrane wrapped microtubule structure. Cell membranes are indicated in yellow.

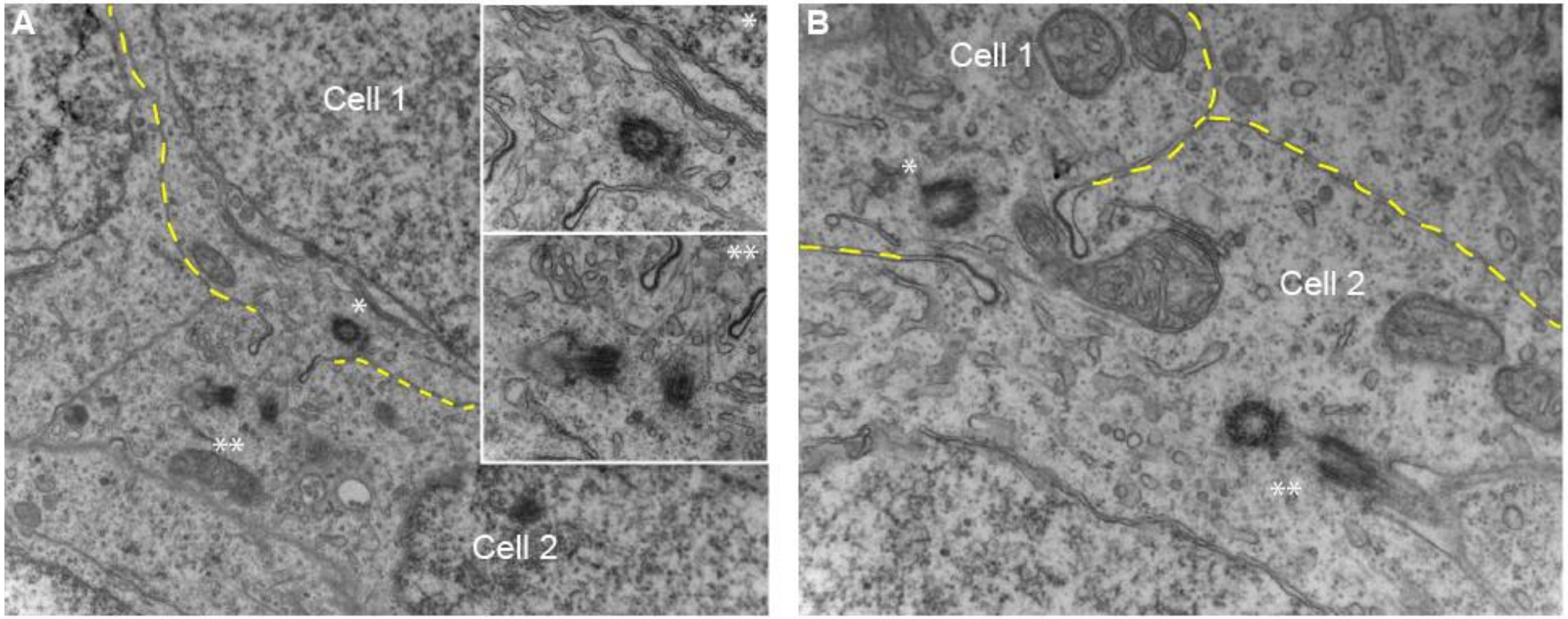

Supplementary Table 1. **Gene Set Enrichment Analysis (GSEA) of rare disruptive mutations, which shows a significant association for familial TGCT with cilia-microtubule function**. Shown are the genes contributing to the leading edge of the top 5 pathways ranked based on enrichment score. Only the cilia-microtubule gene set was significant (*i.e*. *Q* value < 0.1). The genes displayed are those that contribute to the leading edge of each gene set, the value in each cell is the -$\log_{10}$(T1 test *P*-value) used in the ranking for GSEA. Genes with values greater than 1.0 are shown. Please note while gene sets "Reactome loss of nlp from mitotic centrosomes" and "Reactome recruitment of mitotic centrosome" do not differ in terms of leading edge genes, they are different gene sets overall, with the latter being a larger set and hence the association is less significant.

	**Q**	*DNAAF1*	*DRC1*	*MAP4*	*DYNC2H1*	*CEP290*	*CNTRL*	*DYNC1H1*	*HSP90AA1*	*CEP135*	*ALMS1*	*CDK5RAP2*	*MIS12*	*CENPE*	*SRPK1*	*PDS5B*	*PAPD7*	*GSTM4*	*GSTO1*	*GSS*
Cilia-microtubule function	0.01	2.8	2.3	1.8	1.7	1.4														
Reactome loss of nlp from mitotic centrosomes	0.11					1.4	2.6	1.8	1.7	1.4	1.1	1.1								
Reactome recruitment of mitotic centrosome proteins and complexes	0.34					1.4	2.6	1.8	1.7	1.4	1.1	1.1								
Chromosome segregation	0.37												1.9	1.8	1.7	1.7	1.4			
Reactome glutathione conjugation	0.34																	3.9	1.4	1.1

Supplementary Table 2. ***DNAAF1*** **missense allele frequencies from Basten et al. (2013).**

**Mutation**	**ExAC Minor Allele Frequency**	**UK Control Minor Allele Frequency**	***P*-Value for association with TGCT in UK Study**
Gln307Glu	2.0%	2.4%	0.8
Asp435Asn	2.5%	2.8%	0.1
Thr590Met	1.0%	2.1%	0.3

Supplementary Table 3. Primer sequences used to confirm rare disruptive variants in cilia microtubule genes in germline samples and to examine for loss of heterozygosity in tumour samples.

**Oligo sequence (5'-3')**	**Oligo name**	**Gene**
AAGGAAAGCTTTGAGGCCAA	DNAAF1_1F	*DNAAF1*
AGGCGGTGACAGTAGCAG	DNAAF1_1R	*DNAAF1*
TGCCTTTATCGTGCCTATCAG	DNAAF1_1F	*DNAAF1*
GGAACATTTTACTTTCAAGTTTAGGA	DNAAF1_1R	*DNAAF1*
GTCACTCCCTGTGCTGGAAA	DNAAF1_1F	*DNAAF1*
AGTGGTGCAGCATCTCTTTG	DNAAF1_1R	*DNAAF1*
GGGGTTCCTCATAGAGAGCA	DRC1_1F	*DRC1*
AAGGATGTGACAGGGAGCAG	DRC1_1R	*DRC1*
GCACCGAGTTGGTGACAAAT	DRC1_2F	*DRC1*
GAGGGAGGGAGGGAGAAGTT	DRC1_2R	*DRC1*
TTTTTCTAGTTGATCCTGATTTTCTAA	DYNC2H1_1F	*DYNC2H1*
ACCCGCAACATAGCATTGTG	DYNC2H1_1R	*DYNC2H1*
AGGTGGGGGTTCAAGTTAGG	MAP4_1F	*MAP4*
ACACAGTCCCTGGCTCAAAG	MAP4_1R	*MAP4*
TGGTCACAAAAATCAATAAAGAATG	CEP290_1F	*CEP290*
GGTCTTCAAAATGTGAATTTGTT	CEP290_1R	*CEP290*
CCTTTGTTGAACCACCACAACT	CEP290_2F	*CEP290*
GGTGGGAGAATTGCTTGA	CEP290_2R	*CEP290*
AACTTACCAATTTTCCCAATAAGA	LRRC6_1F	*LRRC6*
TTGAACAATGGGGACAGAAA	LRRC6_1R	*LRRC6*
CAAGAGCAGCATGAGGTGAA	CNTRL_1F	*CNTRL*
GCACAGGGTCCTTAGTTTGC	CNTRL_1R	*CNTRL*
AGCCTGGTAGGCAATGTGAT	CNTRL_2F	*CNTRL*
TGGGTACCAACACTGACCTG	CNTRL_2R	*CNTRL*

Supplementary Note 1. The UK Testicular Cancer Collaboration (UKTCC).

Principal Investigator	Study Centre	Study centre address
Rustin, Prof Gordon	Mount Vernon Hospital	Mount Vernon Cancer Centre, Rickmansworth Road, Northwood, Middlesex, HA6 2RN
Srihari, Dr	Royal Shrewsbury Hospital	Trials Unit, Oncology Department, Mytton Oak Road, Shrewsbury, SY3 8XB
Cole, Dr David	Great Western Hospital	3rd Floor, Osprey Unit, Swindon, Wilts, SN3 6BB
Askill, Dr Colin & Bertelli, Dr Gianfilippo	Singleton Hospital and Morriston Hospital	SWW Cancer Institute, Sketty, Swansea, SA2 8QA
Barber, Dr James	Velindre Hospital	Clinical Trials Unit, Velindre Cancer Centre, Velindre Road, Whitchurch, Cardiff CF14 2TL
Gilby, Dr Ed	Royal United Hospital	Dept of Oncology and Haematology, Combe Park, Bath, BA1 3NG
Huddart, Dr Robert	Royal Marsden Hospital Sutton	Downs Rd, Sutton, SM2 5PT
White, Dr Jeff	Beatson Oncology Centre	Beatson West of Scotland Cancer Centre, 1053 Great Western Road, Glasgow, G11 0YN

Braybrooke, Dr Jeremy	Bristol Haematology & Oncology Centre	United Bristol Healthcare NHS trust, Horfield Rd, Bristol, BS2 8ED
Leahy, Dr M and Welch, Dr R	Christie Hospital	Wilmslow Road, Withington, Manchester, M20 4BX
Chakraborti, Dr P	Derbyshire Royal Infirmary	Derby Hospitals NHS Trust, London Road, Derby, DE1 2QY
Joffe, Dr J	St James Hospital Leeds	Dept of Medical Oncology, Leeds, LS9 7TF
Brown, Dr Richard	Wexham Park Hospital	Cancer Clinical Trials, John Ulster Post Grad Centre, Slough, Berks, SL2 4HL
Faust, Dr Guy	Leicester Royal Infirmary	LNR Cancer Reseach Network, Knighton St, Leicester LE1 5WW
Simmonds, Dr Peter	Southampton General Hospital	Cancer Care Directorate, Medical Oncology, Mailpoint 306, Southampton General Hospital, Tremona Rd, SO16 6YD
Mazhar, Dr danish	Addenbrookes Hospital	Addenbrookes Hospital, Cambridge Clinical Trials Centre, Oncology Clinical Trials, (S4) Box 279, Hills Rd, CB2 0QQ
Stockdale, Dr A & Hrouda, Dr D & Humber, Dr C.	University Hospital Walsgrave	Arden Cancer Centre, West Wing, UHCW NHS trust, Clifford Bridge Rd, Coventry, CV2 2DX
Appel, Dr Wiebke	Royal Preston Hospital	Dept of Oncology, Royal Preston Hospital, Sharoe Green Lane North, Fulwood Preston, PR2 9HT
Hong, Dr Anne	Royal Devon & Exeter	Exeter Oncology Centre, Royal Devon and Exeter Hospital, Barrack Rd, Exeter EX2 5DW

Dr Howard	Western General Hospital	Scottish Cancer Research Network, Oncology Admin Corridor, Edinburgh Cancer Centre, Western General Hospital, Crewe Rd South, Edinburgh, EH4 2XU
Dr Fiona Douglas	Freeman Hospital	Clinical Trials Unit, Newcastle General Hospital, Westgate Rd, Newcastle-upon Tyne, NE4 6BE
Bllomfield, Dr David	Royal Sussex County Hospital	Brighton and Sussex University Hospitals, The Sussex Cancer Centre, The Royal Sussex County Hospital, Eastern Road, Brighton, BN2 5BE
Dr Mohammad Butt	Castle Hill Hospital	Castle Hill Hospital, Castle Road, Cottingham HU16 5JQ
Dr Kay Kelly	Raigmore Hospital	Raigmore Hospital, Old Perth Road, Inverness, IV2 3UJ
Dr R Mehra	New Cross Hospital	Greater Midlands Cancer Research Network, The Chestnuts, The Royal Wolverhampton Hospitals, New Cross Hospital NHS Trust, Wednesfield Road, Wolverhampton, WV10 0QP
Dr Richard Brown/Dr Paul Rogers	Royal Berkshire Hospital	Royal Berkshire Hospital, Berkshire Cancer Centre, London Road, Reading, Berkshire, RG1 5AN
Chakraborti, Dr P	Queen's Hospital Burton	Queens Hospital, Burton upon Trent, Belvedere Road, Burton, DE13 0RB
Dr Matthew Hatton	Weston Park Hospital	Consultant Clinical Radiologist. Sheffield Teaching Hospitals NHS Foundation Trust, 8 Beech Hill Road, Sheffield S10 2SB

Hennig, Dr Ivo	Nottingham City Hospital	Nottingham University Hospitals NHS Trust, City Hospital campus, Hucknall Road, Nottingham, NG5 1PB
Dr J McAteer	Belfast City Hospital	Northern Ireland Cancer Centre, Belfast City Hospital, Lisburn Rd, Belfast, BT9 7AB
Dr Savage/Dr Seckl	Charing Cross Hospital	Dept of Medical Oncology,Charing Cross, Fulham, Palace Rd, London W6 8RF
Dr Joanna Gale	Portsmouth Haematology & Oncology Centre	Level B Queen Alexandra Hospital, Cosham, Portsmouth, PO6 3LY
Rustin, Prof Gordon	Hillingdon Hospital	R&D Office - Education Centre, Hillingdon Hospital, Pield Heath Road, Hillingdon, UB8 3NN
Prof Peter Clark	Royal Liverpool & Broadgreen Hospitals	Prescot Street Liverpool, L78XP
Dr Steve Woby	Royal Oldham Hospital/Pennine Acute Hospital	Roachdale Road Oldham OL1 2JH
Dr Adrian Rathmell	James Cook Hospital	Middlesbrough TS4 3BW
Dr Alan Lamont	Colchester/Essex County Hospital	Essex County
Dr Guy Faust	Northampton General	Cliftonville, Northampton NN1 5BD

Dr Naveed Sarwar	Basildon Hospital	Nethermayne Basildon Essex SS16 5NL
Prof Nick Stuart	Glan Clwyd Hospital and Ysbyty Gwynedd	NW Cancer Treatment Centre, Glan Clwyd Hospital, LL18 5UJ
Dr Simon Chowdhury	Guys & St Thomas's	St Thomas Street, London SE1 9RT
Dr Sharon Beesley	Maidstone and Tunbridge NHS Trust	Maidstone Hospital, Hemitage Lane, Barming, Maidstone, Kent ME16 9QQ
Dr Winkler	West Middlesex University Hospital	West Middlesex University Hospital NHS Trust, R&D Department, 4th Floor, East Wing,Twickenham Road, Isleworth Middlesex TW7 6AF
Dr Abdel Hamid	Broomfield Hospital	Broomfield Hospital, West Wing 2, Court Road, Broomfield, Chelmsford, Essex CM1 7ET
Dr Sanjeev Pathak	Doncaster Royal Infirmary	Joint Research Office of Doncaster and Bassetlaw Hospitals NHS Foundation Trust, First Floor 'C' Block, Doncaster Royal Infirmary, Armthorpe Road, Doncaster DN2 5LT
Dr Krishnaswamy Madhavan	Southend University Hospital NHS Foundation Trust	Pittlewell Chase, Westcliff-On-Sea, Essex SSO 0RH
Dr Martin Highley	Derriford Hospital (Plymouth)	Plymouth Hospitals NHS Trust, Derriford Hospital, Plymouth, PL6 8DH
Dr Julian Money-Kyrle	Royal Surrey County Hospital	Royal Surrey County Hospital, St Lukes Cancer Centre, Egerton Road, Guildford, Surrey GU2 7XX

Dr Cathryn Brock	Chelsea & Westminster Hospital NHS Foundation Trust	Chelsea & Westminster Hospital, Unit 101, 1st Floor, Harbour Yard, Chelsea Harbour, London SW10 0XD
Dr Thiagarajan Sreenivasan	United Linconshire Hospitals NHS Trust	Lincoln County Hospital, Greetwell Road, Lincoln, LN2 5QY
Dr Thiagarajan Sreenivasan	United Linconshire Hospitals NHS Trust	Pilgrim Hospital, Boston, Lincolnshire PE21 9QS

Supplementary Note 2. International Testicular Cancer Linkage Consortium (ITCLC) centers from which samples were used in this study.

Investigators	Study Centre	Study centre address
D. Timothy Bishop, Gillian P. Crockford	St James's University Hospital, Leeds	Cancer Research UK Clinical Centre, St James's University Hospital, Leeds, UK
Michael R. Stratton, Elizabeth A. Rapley, Rachel Linger, Sarah Hockley, Darshna Dudakia, Lola Johnson	The Institute Of Cancer Research	Section of Cancer Genetics, Institute Of Cancer Research, 15 Cotswold Road, Sutton, Surrey SM2 5NG, UK
Robert Huddart	The Institute Of Cancer Research/The Royal Marsden Hospital	Academic Radiotherapy Unit, Institute of Cancer Research, Sutton, Surrey, UK
Kathy Tucker, Michael Friedlander	New South Wales and Prince of Wales Hospital	Department of Medical Oncology, Division of Medicine, University of New South Wales and Prince of Wales Hospital Randwick, Sydney Australia
Kelly-Anne Phillips	Peter MacCallum Cancer Centre	Department of Haematology and Medical Oncology, Peter MacCallum Cancer Centre, East Melbourne, Victoria, Australia,
David Hogg, Michael A.S. Jewett	Princess Margaret Hospital	Princess Margaret Hospital and University of Toronto, Toronto, ON, Canada
Radka Lohynska	University Hospital	Department of Radiotherapy and Oncology, University Hospital, Prague, Czech Republic
Gedske Daugaard	Rigshospitalet	Department of Oncology, Rigshospitalet, Copenhagen, Denmark
Stéphane Richard	Centre Expert National Cancers Rares PREDIR	Génétique Oncologique EPHE-UMR 8125 Faculté de Médecine Paris-Sud and Service d'Urologie, CHU, Le Kremlin-Bicêtre, France
Agnes Chompret	Institut Gustave Roussy	Génétique Oncologique, Institut Gustave Roussy, Villejuif, France
Catherine Bonaïti-Pellié	Hôpital Paul Brousse	INSERM U535, Hôpital Paul Brousse, Villejuif, France
Axel Heidenreich	Phillips University	Department of Urological Oncology, Phillips University, Marburg, Germany
Peter Albers	Klinikum Kassel GmbH	Department of Urology, Klinikum Kassel GmbH, Moenchebergstr. 41-43, D-34125 Kassel, Germany
Edith Olah, Lajos Geczi, Istvan Bodrogi	National Institute of Oncology	Department of Molecular Genetics and Department of Chemotherapy, National Institute of Oncology, Budapest, Hungary
Wilma J. Ormiston, Peter A. Daly	St James's Hospital	Department of Medical Oncology, St James's Hospital, Dublin, Ireland
Parry Guilford	University of Otago	Cancer Genetics Laboratory, University of Otago, Dunedin, New Zealand

Sergei A. Tjulandin, Ludmila Liubchenko	N.N. Blokhin Russian Cancer Research Center	Laboratory of Clinical Genetics, Institute of Clinical Oncology, N.N. Blokhin Russian Cancer Research Center, Moscow, Russian Federation
Hans Stoll, Walter Weber	University Hospital	Medical Oncology, University Hospital, Basel, Switzerland
David Forman	Cookridge Hospital	Cancer Epidemiology, University of Leeds, Cookridge Hospital, Leeds LS16 6QB, UK
Timothy Oliver	Barts and The London Queen Mary's School of Medicine	Department of Medical Oncology, Barts and The London Queen Mary's School of Medicine, London, UK
Douglas F. Easton	Cancer Research UK	Cancer Research UK Genetic Epidemiology Unit, Strangeways Research Laboratory, Cambridge, UK
